# Synthesis of Vitamin B3 through a Heterogeneous Photocatalytic Approach Using Metal-Free Carbon Nitride-Based Catalysts

**DOI:** 10.3390/molecules27041295

**Published:** 2022-02-15

**Authors:** Raquel A. Fernandes, Maria J. Sampaio, Joaquim L. Faria, Cláudia G. Silva

**Affiliations:** 1 ALiCE–Associate Laboratory in Chemical Engineering, Faculty of Engineering, University of Porto, Rua Dr. Roberto Frias, 4200-465 Porto, Portugal; raquel.fernandes@fe.up.pt (R.A.F.); mjsampaio@fe.up.pt (M.J.S.); jlfaria@fe.up.pt (J.L.F.); 2 LSRE-LCM – Laboratory of Separation and Reaction Engineering-Laboratory of Catalysis and Materials, Faculty of Engineering, University of Porto, Rua Dr. Roberto s/n, 4200-465 Porto, Portugal

**Keywords:** Vitamin B3, carbon nitride, photocatalysis, organic synthesis

## Abstract

Vitamin B3 (nicotinic acid, VB3) was synthesized through the photocatalytic oxidation of 3-pyridinemethanol (3PM) under visible-light-emitting diode (LED) irradiation using metal-free graphitic carbon nitride (GCN) - based materials. A bulk (GCN) material was prepared by a simple thermal treatment using dicyandiamide as the precursor. A post-thermal treatment under static air and nitrogen flow was employed to obtain the GCN-T and GCN-T-N materials, respectively. The conditions adopted during the post-treatment revealed differences in the resulting materials’ morphological, electronic, and optical properties. The post-treated photocatalysts revealed an enhanced efficiency in the oxidation of 3PM into VB3, with the GCN-T-N photocatalyst being the best-performing material. The defective surface, reduced crystallinity, and superior photoabsorption of GCN-T-N account for this material’s improved performance in the production of VB3. Nevertheless, the presence of nitrogen vacancies in the carbon nitride structure and, consequently, the creation of mid-gap states also accounts to its highly oxidative ability. The immobilization of GCN-T-N in sodium alginate hydrogel was revealed as a promising strategy to produce VB3, avoiding the need for the photocatalyst separation step. Concerning the mechanism of synthesis of VB3 through the photocatalytic oxidation of 3PM, it was possible to identify the presence of 3-pyridinecarboxaldehyde (3PC) as the intermediary product.

## 1. Introduction

Vitamin B3 (or nicotinic acid) is a water-soluble vitamin that acts as a building block in the synthesis of nicotinamide-adenine-dinucleotide (NAD), playing an essential role in the redox reactions occurring in living cells [1]. The primary application of vitamin B3 is in medicine—namely, in treating pellagra disease and alcoholism [2]—leading to an annual production of more than 22,000 tons worldwide [1]. Currently, most vitamin B3 is chemically produced by the ammoxidation and oxidation of pyridines under high pressure and alkali conditions, with a massive energetic demand and the generation of hazardous effluents [1,3]. Heterogeneous photocatalysis has been explored as a valuable and more sustainable alternative to the conventional synthesis processes, using 3-pyridinemethanol as a starting molecule. Spasiano et al. [3] used titanium dioxide (TiO_2_) photocatalyst to convert 3-pyridinemethanol into vitamin B3 without oxygen under a high-pressure UV lamp, achieving a 30% conversion and 87% selectivity after 0.75 h of irradiation. Using TiO_2_ nanotubes, Yurdakal et al. [4] reached a 15% conversion and 35% selectivity in the synthesis of vitamin B3 under an oxygenated atmosphere and a fluorescent lamp light source. The same research group registered an increase in the selectivity (45%) by doping home-prepared TiO_2_ with platinum in alkaline conditions (pH 12) after 3 h of irradiation [5]. Alfè et al. [6] reported the use of a heterojunction strategy, combining TiO_2_ and graphene to increase the conversion of the alcohol and achieving a maximum value of 65% after 5 h of irradiation by a mercury lamp under a continuous nitrogen flow. More recently, Sobahi et al. [7] explored the performance of a heterojunction of metal-doped TiO_2_ (Ag@TiO_2_) and carbon nitride under oxygenated conditions. After 9 h of irradiation with a halogen lamp, researchers reached a 100% alcohol conversion and 100% selectivity for the synthesis of vitamin B3.

TiO_2_-based materials have a significant prevalence concerning the photocatalytic synthesis of vitamin B3 owing to their resulting conversion and selectivity. The use of added metal phases creates several concerns: the possibility of leaching and the lack of sustainability arising from the decreasing availability of some used metals. Concerning artificial processes of photocatalyst activation, the traditional use of low-efficiency irradiation sources, such as halogen and fluorescent lamps, severely threatens the energetic sustainability of conventional light-activated processes. Therefore, the replacement of light sources by light-emitting diodes (LEDs) may account for the environmental sustainability of the process by increasing the energy efficiency and, consequently, increasing the effectiveness of irradiation. The use of carbon-based metal-free semiconductors as photocatalysts circumvents the environmental issues associated with the use of metal-based materials. Among several carbon materials, graphite-like carbon nitride (here labeled as GCN) has gained much attention in photocatalytic systems due to its absorption in the visible region of spectra up to c.a. 460 nm, easy synthesis through the thermal condensation of Earth-abundant and low-cost precursors, nontoxicity, and chemical stability [8,9]. However, its low surface area, high recombination rate between electrons (e^−^) and photogenerated holes (h^+^), and limited absorption of visible radiation negatively affect its photocatalytic performance [8,9,10,11,12]. Among all the methods adopted to overcome the drawbacks of GCN, thermal exfoliation has proven to be highly efficient in tuning its optical, morphological, and electronic properties [13,14,15]. The heating process breaks the hydrogen bonds responsible for the aggregation between the layers of GCN, leading to the splitting of graphite-like sheets [14,16,17,18] and introducing defects on the surface of the catalyst [13,19,20], generally resulting in an enhancement in the photocatalytic activity of the resulting materials.

For the first time, the present work reports the use of powder metal-free catalysts to produce vitamin B3 through the oxidation of 3-pyridinemethanol under visible-LED irradiation. Bulk GCN was thermally post-treated under air (resulting in the designated GCN-T) and then under nitrogen atmosphere (GCN-T-N). The thermally modified catalysts were thoroughly characterized to assess the effect of the thermal post-treatment conditions on their properties. Concerning the advantages of immobilized catalysts in an industrial context, an additional study was also performed using a GCN-T-N catalyst immobilized on a sodium alginate substrate.

## 2. Results and Discussion

### 2.1. Characterization of the GCN-Based Photocatalysts

**Figure 1:**
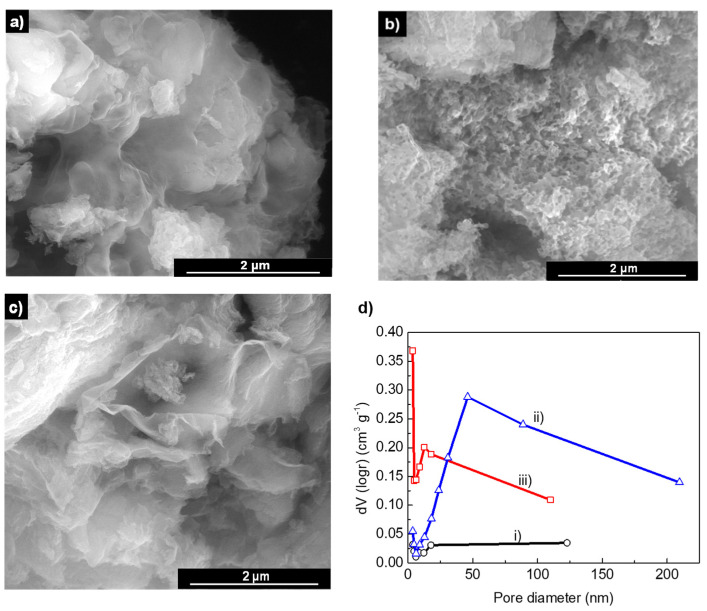
SEM micrographs of (**a**) GCN, (**b**) GCN-T-N, and (**c**) GCN-T. (**d**) Barrett–Joyner–Halenda pore volume distribution derived from the N_2_ adsorption isotherms of (i) GCN, (ii) GCN-T-N, and (iii) GCN-T at −196 °C.

Bulk GCN and thermally post-treated materials under air (GCN-T) and nitrogen (GCN-T-N) atmosphere were morphologically characterized through scanning electron microscopy (SEM). Representative micrographs are presented in Figure 1.

It is reported that bulk GCN (Figure 1a) presents a highly stacked structure, resulting in a low *S*_BET_, which is also observed in this work (7 m^2^ g^−1^, Table 1). In GCN-T-N (Figure 1b), a defective porous surface may be observed, reflected in an increase in its *S*_BET_ (22 m^2^ g^−1^, Table 1). The thickness of the graphite-like layers of GCN-T (Figure 1c) decreases due to the breaking of van der Waals forces and hydrogen bonds during the thermal post-treatment at 500 °C, resulting in a significant increase in the *S*_BET_ (60 m^2^ g^−1^; Table 1) [16,18]. This fact is in line with the pore diameter distribution of GCN-based materials (Figure 1d). The pore diameter of GCN and GCN-T materials seems to be related to the interstices/distance between the carbon nitride layers once these materials did not exhibit pores on their surface. Bulk GCN has an almost negligible pore diameter (Figure 1d), which is in agreement with its compact stacked lamellar structure (Figure 1a).

GCN-T-N presents a broad distribution of pore diameter (from 3.6 nm to 210 nm) and exhibits a high number of larger pores (Figure 1d). In GCN-T, the pore diameter distribution varies from 3.6 nm to 110 nm (Figure 1d).

Defects may occur on the surface of carbon nitride materials due to the presence of nitrogen vacancies in heptazine rings [13,19]. The determination of the C/N ratio obtained from the atomic composition through elemental analysis revealed an increase from 0.56 (GCN and GCN-T, Table 1) to 0.58 (GCN-T-N, Table 1), which may indicate the loss of nitrogen atoms in the aromatic units. XPS analyses revealed a decrease in the atomic content of nitrogen at the catalyst surface for the GCN-T-N compared with bulk GCN (Supplementary Material, Figure S1 and Table S1). This is reflected in the higher C/N ratio observed by XPS for GCN-T-N when compared with the bulk GCN (0.87 and 0.83, respectively), which are in accordance with the results obtained by elemental analysis.

**Figure 2:**
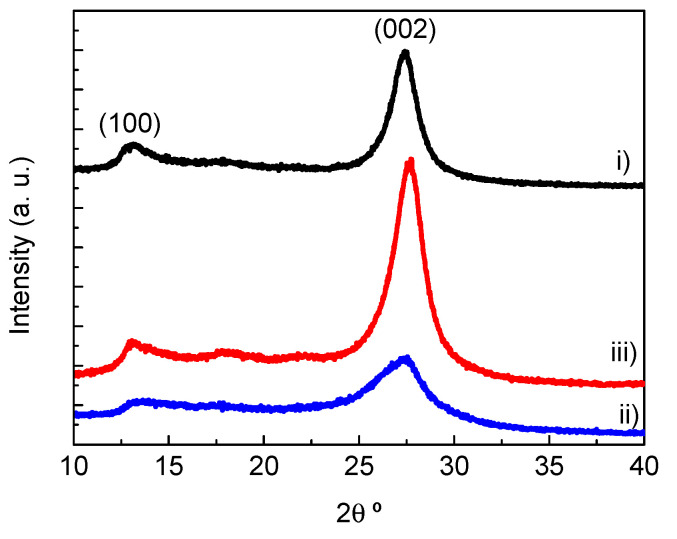
XRD pattern of (i) GCN, (ii) GCN-T-N, and (iii) GCN-T catalysts.

The XRD analysis (Figure 2) confirmed that all GCN-based catalysts exhibit the prominent peaks of carbon nitride materials. The diffraction peak at 27° corresponds to the (002) plane and was ascribed to the distance between the graphitic layers [21,22]. A minor peak was also identified at 13°, attributed to the (100) plane and correlated to the intralayer spacing [21,22]. In the case of GCN-T-N, a sharp decrease was observed in the intensity of both XRD peaks (Figure 2), suggesting a less crystalline structure with no atomic order when compared to bulk GCN and GCN-T, possibly due to its holey surface that creates a highly distorted structure [23]. Additionally, this material exhibited a higher value for the full-width at half-height (FWHM, Table 1) of the (002) peak with relation to bulk GCN and GCN-T, which confirms the decrease in the crystallinity of the material, resulting from the thermal treatment under a nitrogen atmosphere. The high-intensity (002) peak in the XRD spectra of GCN-T follows the previous findings of splitting the carbon nitride layers upon thermal treatment under air. The average crystallite size (d, Table 1) was calculated through the Debye–Scherrer equation [17,24] (see Supporting Data) and followed the order GCN > GCN-T > GCN-T-N, which corroborated the loss of crystallinity in the case of GCN-T-N.

**Figure 3:**
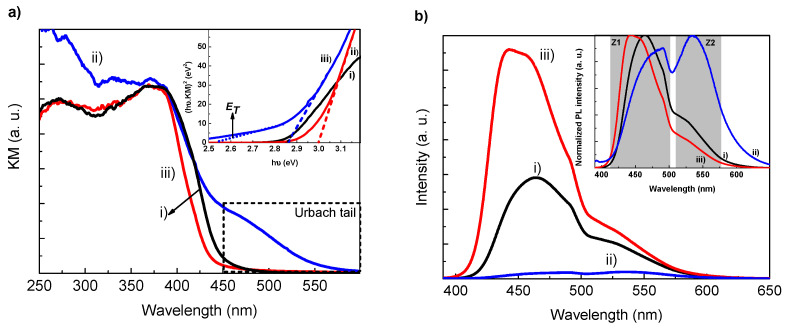
(**a**) DR UV–vis spectra (inset is shown the respective Tauc plots) and (**b**) PL spectra (inset is shown the normalized PL spectra) of (i) GCN, (ii) GCN-T-N, and (iii) GCN-T. Highlighted zones Z1 and Z2 indicate the two PL emission bands.

The optical and electronic properties of GCN-based catalysts were evaluated through DR UV–vis and PL spectroscopies (Figure 3).

As previously reported [11,12,16,18], bulk GCN and GCN-T present similar UV–vis spectra, with an intense absorption band up to c.a. 450 nm (Figure 3a) and a bandgap energy (Eg) of 2.86 eV and 3.00 eV (inset Figure 3a), respectively.

Regarding GCN-T-N, the extension in the absorption up to 550 nm (Urbach tail, Figure 3a) is directly related to the change verified in the color of the catalyst from yellow (GCN) to orange (GCN-T-N).

According to the literature, the presence of an Urbach tail in the DR UV–vis spectra is usually ascribed to n-π* electronic transitions promoted by the existence of mid-gap states located within the bandgap [11,25,26]. These intermediary states of energy may favor the separation of the photogenerated electrons (e^−^) and holes (h^+^), preventing their recombination and allowing the catalyst to absorb photons with energies below its bandgap [11,25,26]. Additionally, Tauc plot analysis (inset Figure 3a) corroborates the existence of additional electronic transitions in GCN-T-N, since it is possible to observe the energy of transition (E_T_, inset Figure 3a) associated with the electronic potential of the mid-gap state (2.54 eV).

In terms of PL, GCN-T displays an intense emission compared to the parent GCN (Figure 3b), which may be attributed to the loss of amino groups during the thermal treatment, promoting defects on the GCN matrix acting as active sites for e^−^/h^+^ recombination [11,26,27,28]. On the other hand, GCN-T-N emission is much reduced compared to bulk GCN (Figure 3b) due to the modified arrangement of C and N atoms in the carbon nitride matrix [23]. A blue shift in the maximum of PL intensity of GCN-T can be observed when compared to bulk GCN (from 463 nm to 444 nm, inset Figure 3b, highlighted zone Z1), which can be attributed to the decrease in the thickness of the carbon nitride layers in the case of GCN-T [27]. Contrariwise, GCN-T-N presents a PL red-shift from 463 nm to 489 nm (inset Figure 3b, highlighted zone Z1) due to the extension of the carbon nitride network when it is subjected to higher temperatures, with the π states hybridizing into a broad state [10]. This band of PL emission is usually ascribed to the recombination rate of e^−^ and h^+^ in the π-conjugated system of graphite-like structures [11,12,26]. A second PL band related to the n-π* electronic transitions in the lone pairs of electrons of N atoms is also observed at 528 nm (inset Figure 3b, zone Z2) [11,12,26]. According to the literature, distorted structures, such as GCN-T-N, have a positive contribution to the occurrence of this type of electronic transition [11,12,26]. Therefore, the electronic excitation in GCN-T-N may be more effective due to the contributions of the two types of transitions.

### 2.2. Photocatalytic Results

The photocatalytic performance of GCN, GCN-T, and GCN-T-N materials was studied in the selective oxidation of 3-pyridinemethanol (3PM) into Vitamin B3 (VB3) under visible-LED irradiation (*λ*_max_ = 412 nm).

The starting alcohol revealed a high photochemical stability under the irradiation conditions adopted, with no 3PM conversion being observed after 7 h of irradiation in the absence of catalyst (data not shown), which was already expected due to the lack of light absorption of this molecule in the wavelength of irradiation of the visible-LED system (Supplementary Material, Figure S2).

**Figure 4:**
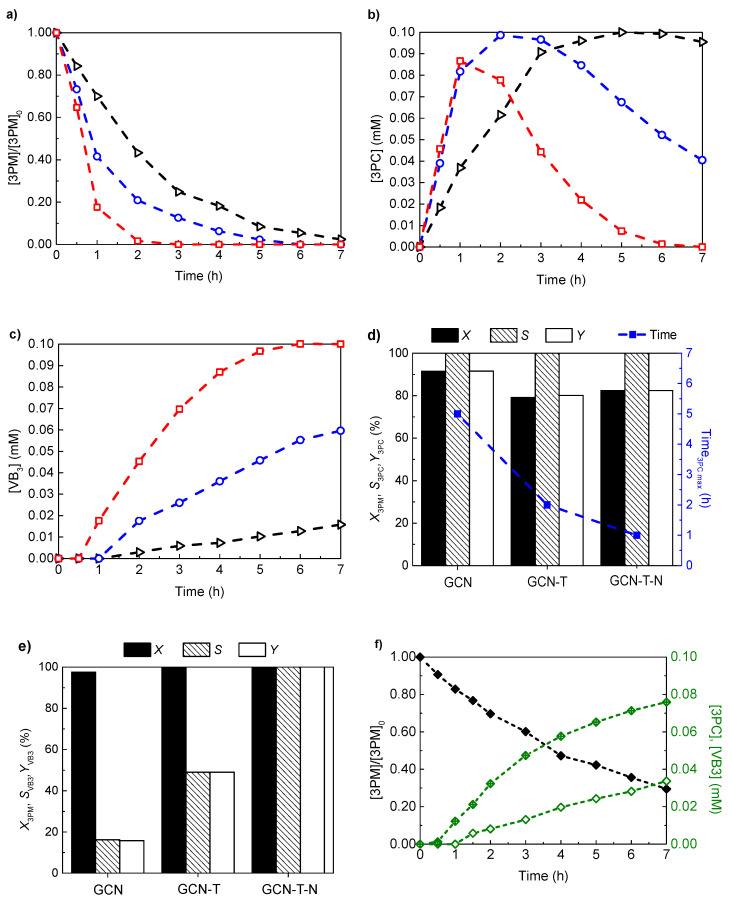
(**a**) Normalized 3PM, (**b**) 3PC, and (**c**) VB3 concentrations over 7 h of irradiation (*λ*_max_ = 412 nm) using GCN (▷), GCN-T-N (□), and GCN-T (○) as catalysts. (**d**,**e**) *X*, *S*, and *Y* obtained for the photooxidation of 3PM into 3PC and VB3 at the maximum of their production, respectively. (**f**) Normalized 3PM (♦), 3PC (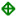), and VB3 (◊) concentrations over 7 h reaction using immobilized GCN-T-N.

As displayed in Figure 4a, the 3-pyridinemethanol (3PM) achieved complete conversion (oxidation) after 3 h using the GCN-T-N photocatalyst. In addition, using the same photocatalyst, the formation of the intermediate 3-pyridinecarboxaldehyde (3PC) with the concomitant production of Vitamin B3 (nicotinic acid, VB3) can be observed in Figure 4b,c, respectively. After 1 h of reaction, the maximum concentration of the intermediate 3PC occurred, then was converted to VB3 (Figure 4c).

This behavior was also observed for bulk GCN and GCN-T photocatalysts. However, the conversion of 3PM showed slower kinetics for both photocatalysts, requiring 3 h for the complete conversion of the starting molecule (3PM).

**Scheme 1:**
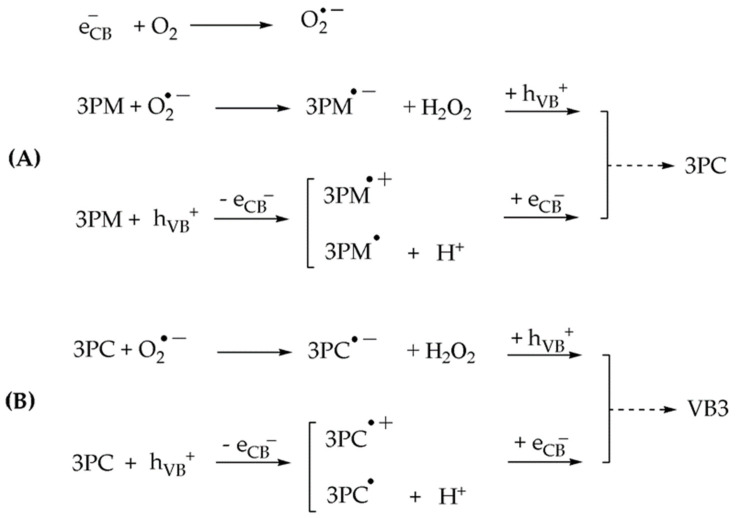
Simplified mechanism of conversion of 3PM into 3PC (**A**) and VB3 (**B**).

In the absence of a strong oxidizing agent, such as dichromate and permanganate oxides, the partial oxidation of primary alcohols usually occurs in two stages: the initial oxidation to the aldehyde, followed by further oxidation to the acid [29]. Figure 4b,c show the 3PC and VB3 concentration profiles obtained during the photocatalytic conversion of the alcohol (3PM). According to previous scavenging studies using similar GCN-based materials, we determined that the main reactive species that participate in the photocatalytic conversion of *p*-anisyl alcohol into p-anisaldehyde under oxygenated conditions were superoxide radicals and photogenerated holes [12]. Based on our previous findings, a simplified mechanism for the sequential conversion of 3PM into 3PC and finally to VB3 is presented as Scheme 1. Briefly, photoexcited electrons occurring at the conduction band react with the adsorbed molecular oxygen O_2_ to generate the O_2_^●−^, which in the presence of the primary alcohol (3PM) produces a charge-separated dehydrogenated radical anion and hydrogen peroxide H_2_O_2_ (Scheme 1A). Oxidative electron transfer to the photogenerated hole of the valence band of the GCN-based catalyst results in the formation of the corresponding aldehyde (3PC). On the other hand, the direct oxidation of the adsorbed alcohol by the photogenerated holes at the valence band of the semiconductor leads to either the formation of an alcohol radical cation or the deprotonated alcohol radical. Either oxidized species adsorbed over the photoexcited GCN material will accept an electron back to produce the aldehyde (3PC). In the present work, the aldehyde is further oxidized into the respective carboxylic acid (VB3, Scheme 1B).

From high-performance liquid chromatography (HPLC) analysis (Supplementary Material, Figure S3) and under the experimental conditions, no other intermediates during the photocatalytic synthesis of VB3 through the oxidation of 3PM and 3PC was detected, suggesting that the GCN-T-N photocatalyst is significantly selective for this type of reaction. In terms of maximum production using GCN-T-N as a photocatalyst, 0.09 mM of 3PC was achieved after 1 h of reaction (Figure 4b) and 0.10 mM of VB3 was achieved after 6 h (Figure 4c), representing a significant improvement when compared to bulk GCN (c.a. 0.01 mM of VB3 produced after 7 h of irradiation).

By determining the photocatalytic parameters of conversion (*X*), selectivity (*S*) and yield (*Y*) in the production of both 3PC and VB3, it was possible to compare the performance of the three materials used in the photocatalytic studies. All catalysts showed a high selectivity (*S* > 99%) for aldehyde production (Figure 4d). Bulk GCN revealed a higher conversion and yield (>95%) than GCN-T and GCN-T-N materials (Figure 4d). However, due to the slower conversion of 3PM when bulk GCN was used as a photocatalyst, the maximum production of 3PC was obtained after 5 h of reaction (Figure 4d). Inversely, GCN-T and GCN-T-N revealed a high performance, reaching c.a. 80% of *X* and *Y* in shorter reaction times (2 h and 1 h, respectively; Figure 4d). This relation was also observed in VB3 production (Figure 4e), with GCN-T and GCN-T-N materials being more efficient than bulk GCN. In fact, after 7 h of reaction, GCN presented a very low *S* (16%) and *Y* (16%) in VB3 production (Figure 4e). In the case of GCN-T, both *S* and *Y* increased to 49% (Figure 4e). GCN-T-N proved to be the best-performing catalyst, achieving 100% of both *S* and *Y* after 7 h of irradiation (Figure 4e).

As previously mentioned, GCN-T-N presents a highly irregular surface (Figure 1c) and decreased crystallinity (Figure 2), caused by the loss of nitrogen atoms (Table 1) during the post-treatment at high temperatures (620 °C) under nitrogen flow. These alterations suggest increased light absorption in the visible range, creating mid-gap states within the bandgap (Figure 3a, Table 1). Moreover, the reduced recombination between e^−^ and the photogenerated h^+^ (Figure 3b) seems to contribute to an efficient photocatalytic process, favoring the conversion of 3PM into 3PC and VB3 when GCN-T-N was used.

It is known that immobilized catalysts have a decreased interaction with target molecules due to mass transfer limitations promoted by the support [27,30]. Nevertheless, immobilization is a strategy used to avoid post-separation operation units after the process, resulting in considerable advantages both from technological and economic points of view. Therefore, the best-performing catalyst (GCN-T-N) was immobilized in a sodium alginate substrate and its performance was evaluated under the same operational conditions adopted for the powdered materials. Results showed that immobilized GCN-T-N achieved a 71% 3PM conversion, with 41% of *S* and 29% of *Y* towards VB3 production after 7 h of irradiation.

The existing reports on the synthesis of VB3 by the photocatalytic conversion of 3PM are compiled in Table 2 and compared to the performance of the system used in the present study.

Most of the studies published deal with the utilization of TiO_2_-based materials as photocatalysts (Table 2, entries 1–5). Sobahi et al. [7] reported the use of g-C_3_N_4_ nanosheets decorated with Ag@TiO_2_ nanospheres for the total conversion of 3PM with a 100% selectivity towards VB3 attained after 9 h of irradiation under air atmosphere using a conventional halogen light source (Table 2, entry 5). Despite the significant achievements reported in the photocatalytic synthesis of VB3, the results obtained in the present work show superior conversions of 3PM (> 98%; Table 2, entries 6–8) compared with the previous ones (Table 2, entries 1–4). Using the thermal post-treated catalysts (GCN-T and GCN-T-N; Table 2, entries 7 and 8), the selectivity was three and six times higher, respectively, than that of bulk GCN (Table 1, entry 6). The best-performing catalyst, GCN-T-N (Table 2, entry 8), achieved similar results to Ag@TiO_2_/g-C_3_N_4_ (Table 2, entry 5) and in less time (7 h instead of 9 h). Moreover, the radiation source adopted in the present study (visible-LEDs) exhibits essential environmental and economic advantages compared to conventional halogen lamps—namely, a relatively long-life span, low cost, and high energy efficiency [31]. The promising results of 3PM conversion and selectivity for VB3 production (71% and 34%, respectively) obtained using the immobilized GCN-T-N (Table 2, entry 9) are favorable, foreseeing a more technological approach by avoiding the costly and time-consuming catalyst particle separation and the possibility of adapting the immobilized catalyst film to different reactor configurations.

## 3. Materials and Methods

### 3.1. Materials

Dicyandiamide (DCN, 99%), calcium chloride (CaCl_2_, >99%), 3-Pyridinemethanol (3PM, 98%), 3-Pyridinecarboxaldehyde (3PC, 98%), and Vitamin B3 (VB3, ≥98%) were purchased from Sigma-Aldrich (Darmstadt, Germany). Formic acid (HCO_2_H, 98%) was acquired from Fluka (Darmstadt, Germany). Methanol (CH_3_OH, 99.6%) and sodium alginate were purchased from VWR^TM^ (Arnhem, The Netherlands). All the reagents were used without further purification. Ultra-pure water (18 MΩ cm^−1^) was obtained from a Millipore Milli-Q water purification system (Darmstadt, Germany).

### 3.2. Synthesis of Metal-Free GCN-Based Catalysts

The bulk GCN material was prepared by thermal decomposition of DCN as described elsewhere [18]. Briefly, 2 g of the precursor DCN was placed in a covered crucible and heated in a muffle to 550 °C for 4 h with a heating rate of 2 °C min^−1^ under a static air atmosphere. The obtained yellow bulk GCN material was ground, washed with deionized water, and dried at 60 °C for 12 h under a static air atmosphere. In a second step, thermal exfoliation was carried out by placing 0.95 g of bulk GCN in an open crucible, followed by a temperature rise to 500 °C using a heating rate of 2 °C min^−1^ in the muffle under static air atmosphere. The temperature was maintained for 2 h, and the resulting material was denoted as GCN-T. For GCN-T-N material synthesis, the bulk GCN was placed into a quartz tubular reactor, heated until 620 °C at 5 °C min^−1^, and the treatment remained for 2 h under an inert atmosphere (nitrogen at 80 mL min^−1^).

The GCN-T-N powder was dispersed on sodium alginate (SA) polymer to immobilize the photocatalyst by adapting the procedure described elsewhere [32]. Concisely, an aqueous solution containing 25 g L^−1^ of SA was magnetically stirred and heated at 50 °C for 2 h. Then, 50 mg of GCN-T-N was added under stirring until a homogeneous dispersion in the SA solution was obtained. To favor the gelation process and the cross-linking between SA and the photocatalyst, the addition of 25 mL of 3% (*w*/*v*) CaCl_2_ aqueous solution was carried out. The final dispersion was stirred for 8 h at 50 °C. Then, the dispersion was placed in a glass plate and spread using a knife film applicator (Elcometer 3580, Warren, USA). The obtained flat film (40 mm × 50 mm, with 5 mm of thickness) was washed and stored in deionized water until further use.

### 3.3. Photocatalyst Characterization

The morphology of the materials was assessed by scanning electron microscopy (SEM) in an FEI Quanta 400 FEG ESEM/EDAX Genesis X4M (15 keV) instrument (Hillsboro, USA). The chemical composition of the materials was determined by elemental analysis. Carbon, nitrogen, and hydrogen were determined in a Vario micro cube analyzer (Elementar GmbH, Frankfurt, Germany) by the combustion of the sample at 1050 °C. Each sample was analyzed in triplicate. X-ray diffraction (XRD) analysis was carried out in a PANalytical X’Pert MPD instrument (Malvern, United Kingdom) equipped with an X’Celerator detector and secondary monochromator (Cu Ka k = 0.154 nm, 50 kV, 40 mA; data recorded at a 0.017° step size, 100 s/step). X-ray photoelectron spectroscopy analyses (XPS) were carried out in a Kratos AXIS Ultra HAS equipment and Al monochromator operating at 15 kV (90 W, Manchester, United Kingdom) in hybrid mode. The obtained spectra were analyzed and deconvoluted using the CasaXPS software. The diffuse-reflectance UV–Vis spectra (DR UV–Vis) of the materials were obtained on a JASCO V-560 UV–Vis spectrophotometer equipped with an integrating sphere attachment (JASCO ISV-469, Pfungstadt, Germany). The bandgap of the photocatalysts was determined from the respective Tauc plot. The Brunauer–Emmett–Teller (BET) specific surface area (*S*_BET_) was determined through the N_2_ adsorption data at −196 °C in the relative pressure range 0.05–0.20, using a Quantachrome NOVA 4200e apparatus (Boynton Beach, USA). Solid-state photoluminescence (PL) spectra were obtained at room temperature on a JASCO (FP 82000) fluorescence spectrometer (Pfungstadt, Germany) equipped with a 150W Xenon lamp as a light source using an excitation and emission bandwidth fixed at 2.5 nm. The excitation wavelength was set at 370 nm and the emission was measured in the 380–650 nm range.

### 3.4. Photocatalytic Experiments

The photocatalytic efficiency of GCN, GCN-T, and GCN-T-N catalyst materials was studied in the selective oxidation of 3-pyridinemethanol (3PM) into Vitamin B3 (VB3) under visible-LED irradiation (*λ*_max_ = 412 nm). All experiments were carried out in a cylindrical borosilicate reactor filled with 0.10 mM aqueous solution of 3PM at 20 °C and a catalyst load of 1 g L^−1^. The experiments were performed under continuous magnetic stirring and oxygen saturation (continuous airflow), using a four visible LED system (*λ*_max_ = 412 nm) as the radiation source. The average irradiance of each LED was 49.8 mW cm^−2^, as determined by using a UV–vis spectroradiometer (OceanOptics USB2000 +, Ocean Insight, Duiven, Netherlands). A dark period of 30 min was initially established to reach the adsorption–desorption equilibrium. The suspension was irradiated for 7 h and the 3PM, 3PC, and VB3 concentrations were monitored by High-Performance Liquid Chromatography (HPLC) using a Shimadzu Corporation apparatus equipped with a Diode Array Detector (SPD M20A, Kyoto, Japan). A Surf C18 AQ 100A 3 µm (100 × 2.1mm) column was used with a solvent delivery pump (LC-30AD) at a flow rate fixed at 0.25 mL min^−1^. The temperatures of the column oven and autosampler were set at 40 °C and 4.0 °C, respectively. An equilibrated mixture of acetonitrile (A) and 40 mM ammonium acetate (B) with a ratio of 2:98 (A:B) was isocratically eluted for 15 min. The 3PM, 3PC, and VB3 concentrations were determined at 260 nm, 230 nm, and 215 nm, respectively.

According to the chemical stoichiometry of the oxidative reaction, one molecule of 3PM can be oxidized to one molecule of 3PC, which is consequently oxidized to VB3 (1:1:1). The synthesis of 3PC and VB3 only starts when the radiation source is turned on, which means that the initial concentration of both molecules (Z_0_) is zero. The performance of the reaction was evaluated in terms of 3PM conversion (*X*), selectivity (*S*), and yield (*Y*) towards the 3PC and VB3 production (see Supporting Data).

Regarding the immobilized GCN-T-N, the polymeric material was placed in the photoreactor with a cylindrical shape around its inside walls (Supplementary Material, Figure S4. The photocatalytic reaction was carried out under the same reaction conditions as the powdered catalysts.

## 4. Conclusions

GCN-based photocatalysts obtained through its thermal post-treatment under air (GCN-T) and nitrogen (GCN-T-N) atmospheres were successfully applied in the synthesis of vitamin B3 (VB3) through the oxidation of 3-pyridinemethanol (3PM) under visible-LED radiation (*λ*_max_ = 412 nm). The different conditions applied in the post-treatment to bulk GCN led to less crystalline materials with distinct morphological, optical, and electronic properties.

The oxidation of the starting alcohol (3PM) resulted in the formation of the respective aldehyde (3-pyridinecarboxaldehyde; 3PC) and the nicotinic acid (VB3).

The thermal post-treatment proved to positively impact the photocatalytic performance of the modified catalysts (GCN-T and GCN-T-N) compared to bulk GCN. GCN-T-N was revealed to be highly efficient, achieving a 100% 3PM conversion and selectivity towards VB3 production after 7 h of irradiation, corresponding to the best result among all the catalysts already reported in the literature.

The highly defective surface of GCN-T-N, with reduced crystallinity and improved photo absorption, contributes to enhancing the photocatalytic activity compared to the other tested materials. The irregular structure caused by the presence of nitrogen vacancies in the heptazine rings led to the creation of mid-gap states within the bandgap of the catalyst, which improved its oxidative ability.

The study involving the use of immobilized GCN-T-N revealed a promising potential of this photocatalytic system to be used in real-case applications.

## Figures and Tables

**Table 1:** The atomic ratio between carbon and nitrogen, specific surface area (*S*_BET_), XRD peak (002) width at half height (FWHM), and crystallite size (*d*) of the synthesized catalysts.

Catalyst	*S*_BET_ (m^2^ g^−1^)	C/N Ratio	FWHM (°)	*d* (nm)
GCN	7	0.56	2.09	4.2
GCN-T	60	0.56	2.23	3.9
GCN-T-N	22	0.58	2.54	3.8

**Table 2:** A review of the heterogeneous photocatalytic synthesis of vitamin B3 (VB3) through the conversion of 3-pyridinemethanol (3PM).

Entry	Catalyst	Solvent	Atmosphere	Irradiation System	Reaction Time (h)	*X* (%)	*S* (%)	Ref.
1	TiO_2_	Water	Nitrogen	High-pressure UV lamp	0.75	30	87	[3]
2	TiO_2_ nanotubes	Water	Air	Fluorescent lamp	n.i.	15	35	[4]
3	Pt/TiO_2_	Water	Air	Fluorescent lamp	3	15	45	[5]
4	TiO_2_/graphene	Water	Nitrogen	Mercury lamp	5	63	35	[6]
5	Ag@TiO_2_/g-C_3_N_4_	Water	Air	Halogen lamp	9	100	100	[7]
6	GCN	Water	Air	Vis-LED	7	98	16	Present study
7	GCN-T					100	49	
8	GCN-T-N					100	100	
9	GCN-T-N *					71	34	

n.i.—not indicated; *X*: conversion; *S*: selectivity; * immobilized GCN-T-N.

## Data Availability

Not applicable.

## Supplementary Materials

The following are available online: Debye–Scherrer equation definition; photocatalytic parameters calculation and Figures S1–S4, and Table S1.

## Sample Availability

Not available.
