# Supplementary material for: Synthesis of Vitamin B3 through a Heterogeneous Photocatalytic Approach Using Metal-Free Carbon Nitride-Based Catalysts
Source: Molecules. 2022 Feb 15;27(4):1295. doi: 10.3390/molecules27041295 (PMC8878246; doi:10.3390/molecules27041295)
Supplement: Supplementary file 1 [file molecules-27-01295-s001.zip › molecules-1576959-supplementary.pdf]

Synthesis of vitamin B3 through a heterogeneous photocatalytic approach using metal-free carbon nitride-based catalysts

Raquel A. Fernandes†, Maria J. Sampaio†, Joaquim L. Faria, Cláudia G. Silva*

Laboratory of Separation and Reaction Engineering – Laboratory of Catalysis and Materials (LSRE-LCM), Department of Chemical Engineering, Faculty of Engineering, University of Porto, Rua Dr. Roberto Frias, s/n, 4200-465 Porto, Portugal

*Corresponding author e-mail address: cgsilva@fe.up.pt (C.G. Silva)

† These authors contributed equally to this work.

Supplementary Material

Equation (S1)–Debye-Scherrer equation definition.

Equations (S2)–(S4)–Photocatalytic parameters calculation.

Figure S1–C1s and N1s XPS spectra of GCN and GCN-T-N samples.

Figure S2–DRUV-Vis recorded in Kubelka-Munk units (a.u.) of GCN (a), GCN-T (b) and GCN-T-N (c); and UV-vis spectra in absorbance units (cm^{-1}) of 3PM (i), 3PC (ii) and VB3 (iii) in left y-axis. LEDs system intensity (a.u.) in the right y-axis.

Figure S3–High-Performance Liquid Chromatography (HPLC) chromatograms of 3-pyridinemethanol (3PM), 3-pyridinecarboxaldehyde (3PC) and (nicotinic acid, VB3) compounds at 260 nm, 230 nm, and 215 nm, respectively. Initial conditions: 0.10 mM aqueous solution of 3PM and GCN-T-N as photocatalyst.

Figure S4–Scheme of the photocatalytic reactor using immobilized GCN-T-N for VB3 synthesis.

Table S1–Carbon and nitrogen atomic content, C/N ratio and N1/N2 peak ratio of GCN and GCN-T-N obtained through XPS analysis.

Debye-Scherrer equation definition

The average crystallite size (d, Table 1) was calculated through Debye-Scherrer equation:

$$d=\frac{k\lambda}{\beta cos\theta} \quad \text{(S1)}$$

, where λ is the wavelength of the light used for the diffraction (Cu-Kα radiation), k is the Scherrer constant (0.9), θ is the diffraction angle, and β is the full-width at half maximum (FWHM) of the sharp peaks.

Photocatalytic parameters calculation

The 3PM conversion (X), selectivity (S) and yield (Y) towards the 3PC and VB3 production are then defined as:

$$X\,(\%)=\frac{3PM_0\text{-}3PM_t}{3PM_0}\times 100 \quad \text{(S2)}$$

$$S\,(\%)=\frac{Z_t\text{-}Z_0}{3PM_0\text{-}3PM_t}\times 100=\frac{Z_t}{3PM_0\text{-}3PM_t}\times 100 \quad \text{(S3)}$$

$$Y\,(\%)=\frac{Z_t\text{-}Z_0}{3PM_0}\times 100=\frac{Z_t}{3PM_0}\times 100 \quad \text{(S4)}$$

, which $3PM_0$ is the initial concentration of 3PM (mM), $3PM_t$ is the concentration of 3PM (mM) at a given time t, and Z_t is the concentration of 3PC or VB3 (mM) at a given time t. It is worth mentioning that following the adopted definitions, it results that $Y=X\times S$.

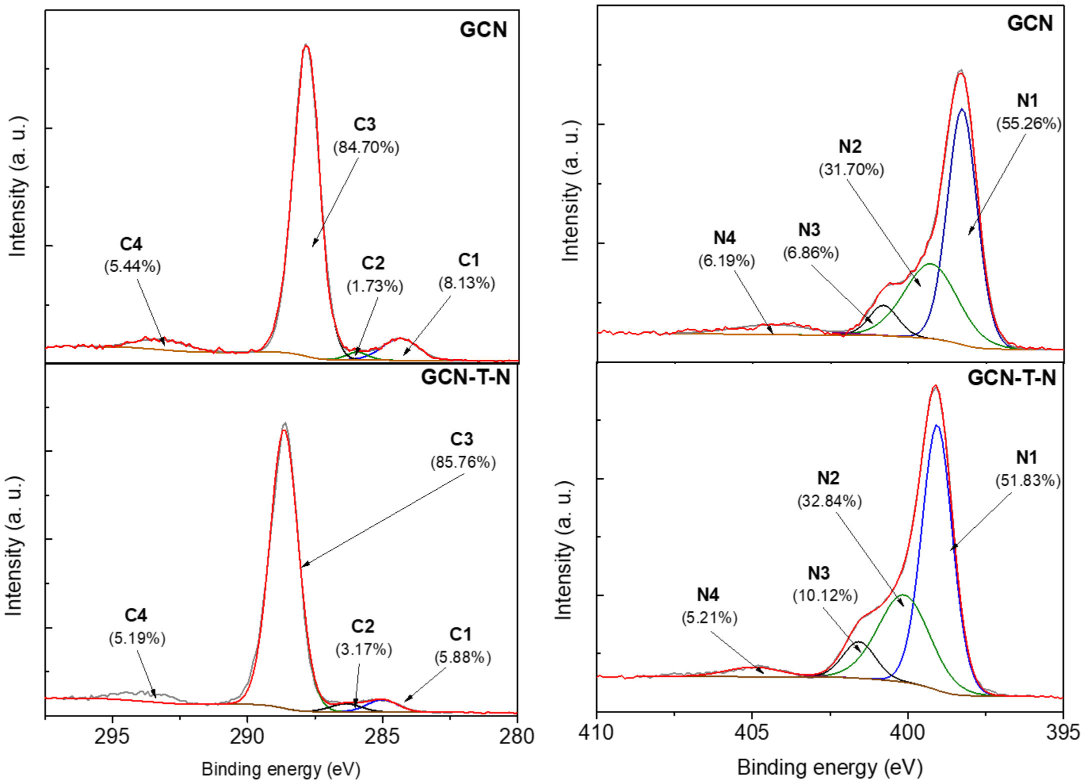

Figure S1–C1s and N1s XPS spectra of GCN and GCN-T-N samples.

Table S1–Carbon and nitrogen atomic content, C/N ratio and N1/N2 peak ratio of GCN and GCN-T-N obtained through XPS analysis.

Material	C (%)	N (%)	C/N ratio	N1/N2
GCN	44.3	53.6	0.83	1.74
GCN-T-N	45.4	52.4	0.87	1.58

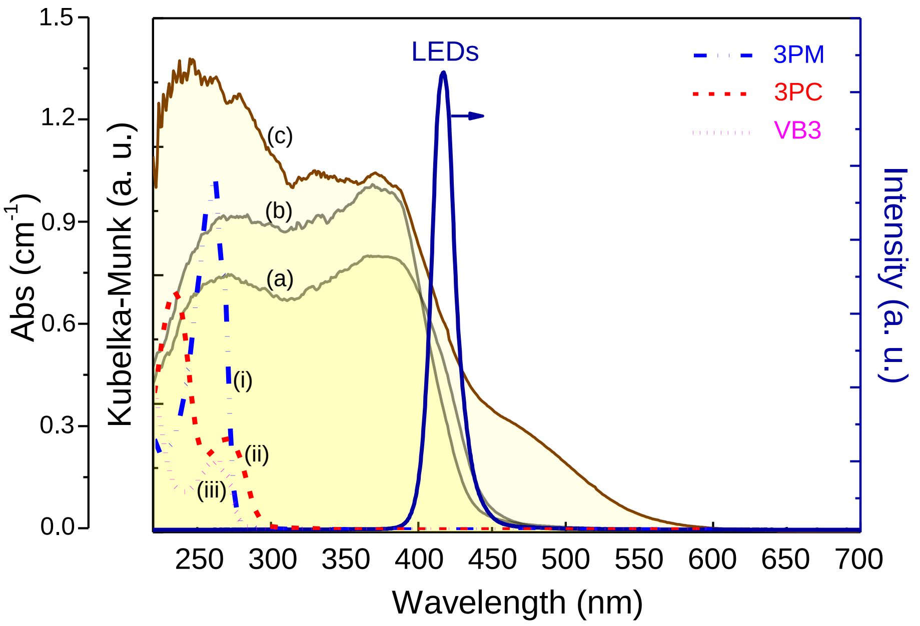

Figure S2–DRUV-Vis recorded in Kubelka-Munk units (a.u.) of GCN (a), GCN-T (b) and GCN-T-N (c); and UV-vis spectra in absorbance units (cm^{-1}) of 3PM (i), 3PC (ii) and VB3 (iii) in left y-axis. LEDs system intensity (a.u.) in the right y-axis.

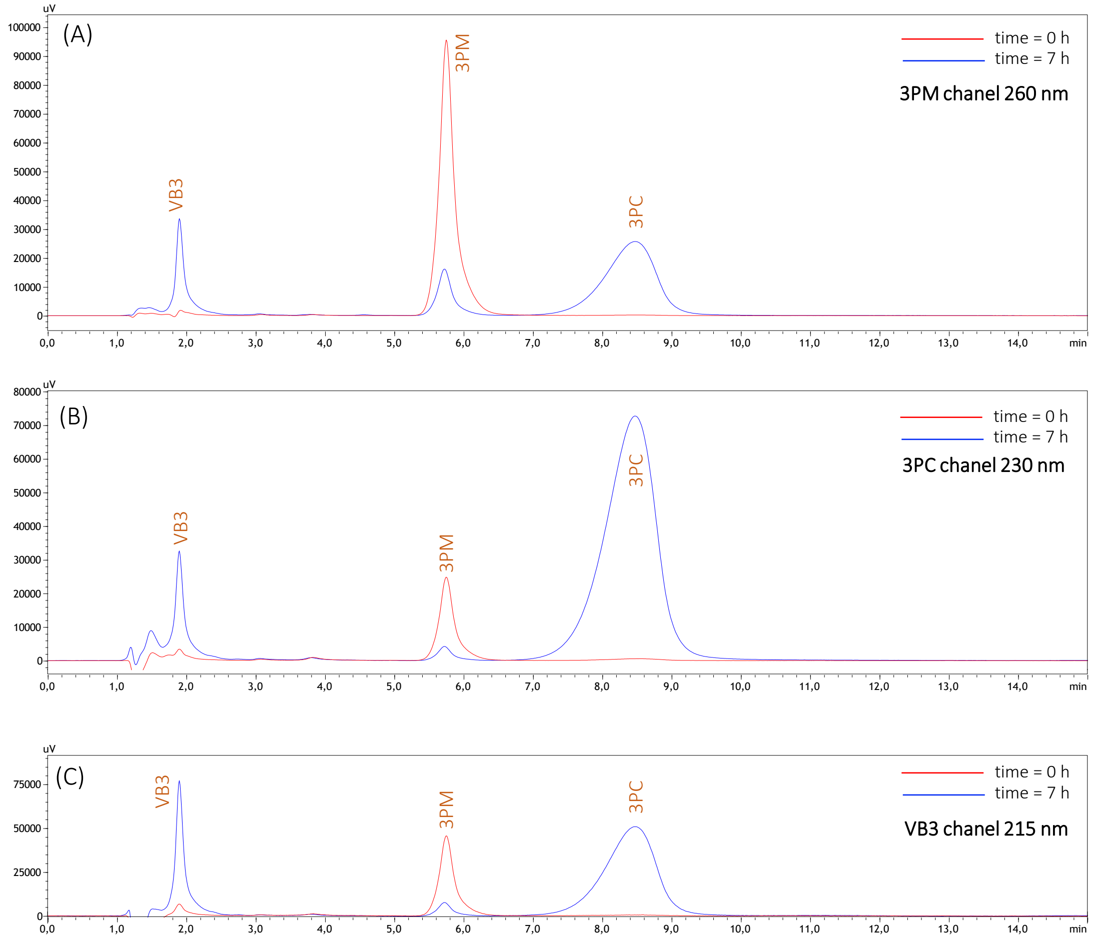

Figure S3–High-Performance Liquid Chromatography (HPLC) chromatograms of 3-pyridinemethanol (3PM), 3-pyridinecarboxaldehyde (3PC) and (nicotinic acid, VB3) compounds at 260 nm, 230 nm, and 215 nm, respectively. Initial conditions: 0.10 mM aqueous solution of 3PM and GCN-T-N as photocatalyst.

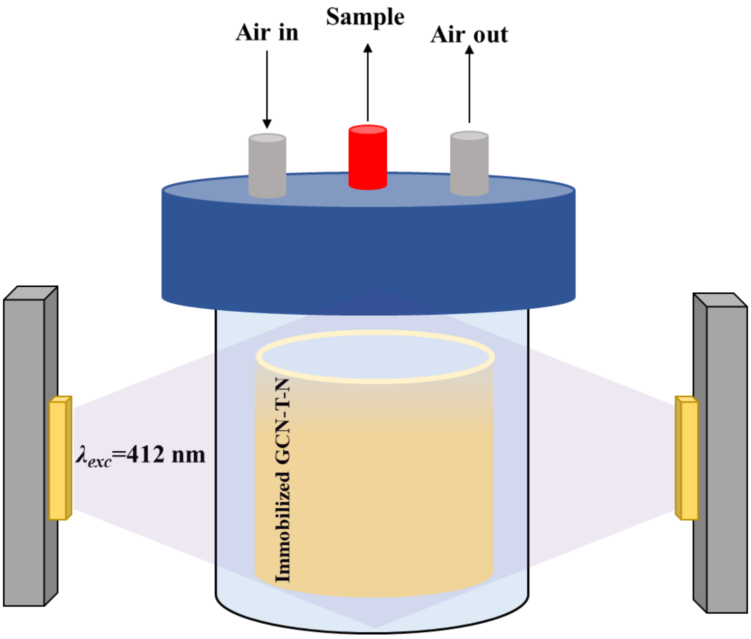

Figure S4–Scheme of the photocatalytic reactor using immobilized GCN-T-N for VB3 synthesis.
